# *AtNPF2.5* Modulates Chloride (Cl^−^) Efflux from Roots of *Arabidopsis thaliana*

**DOI:** 10.3389/fpls.2016.02013

**Published:** 2017-01-05

**Authors:** Bo Li, Jiaen Qiu, Maheswari Jayakannan, Bo Xu, Yuan Li, Gwenda M. Mayo, Mark Tester, Matthew Gilliham, Stuart J. Roy

**Affiliations:** ^1^Australian Centre for Plant Functional GenomicsAdelaide, SA, Australia; ^2^School of Agriculture, Food and Wine, Waite Research Institute, University of AdelaideAdelaide, SA, Australia; ^3^Division of Biological and Environmental Science and Engineering, King Abdullah University of Science and TechnologyThuwal, Saudi Arabia; ^4^ARC Centre of Excellence in Plant Energy BiologyAdelaide, SA, Australia

**Keywords:** *Arabidopsis thaliana*, chloride transport, MIFE, NPF2.5, salinity tolerance, TEVC

## Abstract

The accumulation of high concentrations of chloride (Cl^−^) in leaves can adversely affect plant growth. When comparing different varieties of the same Cl^−^ sensitive plant species those that exclude relatively more Cl^−^ from their shoots tend to perform better under saline conditions; however, the molecular mechanisms involved in maintaining low shoot Cl^−^ remain largely undefined. Recently, it was shown that the NRT1/PTR Family 2.4 protein (NPF2.4) loads Cl^−^ into the root xylem, which affects the accumulation of Cl^−^ in Arabidopsis shoots. Here we characterize NPF2.5, which is the closest homolog to NPF2.4 sharing 83.2% identity at the amino acid level. *NPF2.5* is predominantly expressed in root cortical cells and its transcription is induced by salt. Functional characterisation of NPF2.5 via its heterologous expression in yeast (*Saccharomyces cerevisiae*) and *Xenopus laevis* oocytes indicated that NPF2.5 is likely to encode a Cl^−^ permeable transporter. Arabidopsis *npf2.5* T-DNA knockout mutant plants exhibited a significantly lower Cl^−^ efflux from roots, and a greater Cl^−^ accumulation in shoots compared to salt-treated Col-0 wild-type plants. At the same time, ${\text{NO}}_{3}^{-}$ content in the shoot remained unaffected. Accumulation of Cl^−^ in the shoot increased following (1) amiRNA-induced knockdown of *NPF2.5* transcript abundance in the root, and (2) constitutive over-expression of *NPF2.5*. We suggest that both these findings are consistent with a role for NPF2.5 in modulating Cl^−^ transport. Based on these results, we propose that NPF2.5 functions as a pathway for Cl^−^ efflux from the root, contributing to exclusion of Cl^−^ from the shoot of Arabidopsis.

## Introduction

It has been well documented that for plants such as grapevine (*Vitis* spp.) and citrus (*Citrus* spp.), when grown in saline soils, the accumulation of chloride ions (Cl^−^) in shoot tissues is more commonly associated with a reduction in plant growth and fruit yield than the accumulation of sodium ions (Na^+^) in the shoot (Walker et al., 1997; Storey and Walker, 1999; Munns and Tester, 2008; Teakle and Tyerman, 2010). This negative association between plant salinity tolerance and accumulation of Cl^−^ in leaves has also been shown for varieties of several species generally thought to be more Na^+^ sensitive, such as barley and wheat (Teakle and Tyerman, 2010). Despite the effects of excessive shoot Cl^−^ accumulation, the physiological and genetic control of Cl^−^ movement from the root to the shoot has been rarely studied (Munns and Tester, 2008; Teakle and Tyerman, 2010; Roy et al., 2014; Munns and Gilliham, 2015). This contrasts with our deeper understanding of the regulation of Na^+^ transport that has led to improvements in salt tolerance of both model plants and crops (Gaxiola et al., 2001; Zhang and Blumwald, 2001; Møller et al., 2009; Plett et al., 2010; Munns et al., 2012; Roy et al., 2013).

In order to maintain an optimal growth rate in saline conditions glycophytic crop species often exclude both Cl^−^ and Na^+^ from their shoots - a process termed “ion exclusion” (Munns and Tester, 2008; Teakle and Tyerman, 2010). Cl^−^ exclusion, therefore, has been the focus of studies aimed at improving salinity tolerance of Cl^−^-sensitive species (Brumós et al., 2010; Teakle and Tyerman, 2010; Henderson et al., 2014). Putative mechanisms to prevent Cl^−^ from accumulating to high concentrations in the shoot have been described in several papers (Xu et al., 1999; White and Broadley, 2001; Teakle and Tyerman, 2010) and include: reducing net Cl^−^ uptake from the soil into the root (Britto et al., 2004; Lorenzen et al., 2004); minimizing net Cl^−^ loading in to the transpiration stream (Greenway and Munns, 1980; Teakle et al., 2007; Lauchli et al., 2008); increasing root storage by compartmentation of Cl^−^ in cortical cells (Storey and Walker, 1999; Storey et al., 2003) and partitioning of Cl^−^ into less-sensitive types of cells in leaves (Fricke et al., 1996). Genotypic differences in Cl^−^ efflux from the root of salt sensitive and salt tolerant poplar species demonstrated the contribution of salt-inducible Cl^−^ excretion from the root to plant salinity tolerance (Sun et al., 2009). The exclusion of Cl^−^ has been found to be a multigenic trait (Gong et al., 2011; Long et al., 2013; Genc et al., 2014), and this is not surprising considering the many potential processes that can underpin Cl^−^ transport to the shoot that are detailed above.

Only a few genes encoding transport proteins that affect long-distance Cl^−^ movement in plants have been identified. Chloride Channel-c (CLCc) compartmentalizes Cl^−^ in the vacuoles of roots under saline conditions reducing Cl^−^ transport to the shoot (Jossier et al., 2010). Colmenero-Flores et al. (2007) showed that the cation chloride co-transporter (CCC), which is usually restricted to one member per plant and expressed in the cells that surround the xylem, affects net loading of Cl^−^ and Na^+^ (with K^+^) into the root xylem. However, the finding that AtCCC and *Vitis vinifera* CCC (VviCCC) are localized to the Golgi and *trans*-Golgi network (Henderson et al., 2015), and that *Orysa sativa* CCC (OsCCC) is involved in osmoregulation and tissue growth suggests that the role of CCC in long distance Cl^−^ transport is likely to be indirect. More recently, *AtNPF2.4* (Li et al., 2016) and *AtSLAH1* (*Slow Anion Channel Associated 1 Homolog1*) (Cubero-Font et al., 2016; Qiu et al., 2016) have been identified as genes encoding plasma membrane-localized anion transporters in the root stele that facilitate transfer of Cl^−^ into the root xylem. As one member of the NRT1/PTRs in Arabidopsis, *NPF2.4* belongs to the NAXT (for Nitrate Excretion Transporter) subfamily (a seven-gene clade) that was named after *NAXT1* (*NPF2.7*), a plasma membrane transporter involved in the efflux of ${\text{NO}}_{3}^{-}$ from the root surface (Segonzac et al., 2007; Tsay et al., 2007; Léran et al., 2014). Another NAXT family member, NPF2.3, was identified following functional characterization as a transporter that was more selective for ${\text{NO}}_{3}^{-}$ than for Cl^−^ and was important for root-to-shoot transfer of ${\text{NO}}_{3}^{-}$ (Taochy et al., 2015). *NPF2.3* and *NPF2.4* were both shown to be regulated by NaCl, indicating the possible involvement of the NAXT subfamily in plant salinity responses (Taochy et al., 2015; Li et al., 2016). The other four *NAXT* genes, *NPF2.1, NPF2.2, NPF2.5*, and *NPF2.6*, are yet to be functionally characterized. *NPF2.5* was revealed by a comparative microarray study to be preferentially expressed in the root (Tsay et al., 2007). On the basis of having the smallest phylogenetic distance in protein sequence to the Cl^−^-efflux transporter NPF2.4 (Li et al., 2016), it was hypothesized that NPF2.5 may also function as a Cl^−^ transporter in the root.

## Materials and methods

### Plant material and growth conditions

*Arabidopsis thaliana* seeds of Col-0 background and an *npf2.5* (SM_3.31001) were obtained from the European Arabidopsis Stock Centre (Nottingham, UK). The *npf2.5* knockout mutant contains a transposable dspm-element insertion in the 4th exon of the *NPF2.5* ORF. All plants were grown in a growth room with a photoperiod of 10/14 h (light/dark), the photon irradiance at the level of the plant leaves was 120 μmol m^−2^ s^−1^. Growth room temperature was maintained between 21 and 23°C, and humidity was kept between 60 and 75%.

Hydroponically grown plants: Arabidopsis seeds were germinated and grown in growth rooms using the conditions described above and methods described in Conn et al. (2013). Salt stress was applied to the growth solution 4–5 weeks after germination.

MS-plate grown plants: Arabidopsis seeds were surface sterilized using 70% (v/v) ethanol solution and commercial bleach (Unilever Australasia, Australia) for 10 min each. The bleach residue was removed by rinsing the seeds with dH_2_O at least 5 times. Seeds were placed on ½ MS plates and sealed. Plates were kept in growth rooms described above.

Soil grown plants: the material and protocol used to grow Arabidopsis in soil was described in Møller et al. (2009). Soil grown plants were treated with 150 mM NaCl solution (500 ml per time, 3 times per week). This was done by applying NaCl solution to the bottom of each planting pot (one plant per pot).

### Expression analysis

Transcript abundance analysis by qRT-PCR was performed following the method described in Burton et al. (2008) and Jha et al. (2010). The sequences of primers for determining *NPF2.5* expression were 5′ TCCTGCTCTTGTGATGGTTG 3′ (Forward) and 5′ CACGAGGTGGTTCTTTGGAT 3′ (Reverse). To test the primer specificity, melt curve analysis was performed and the PCR products were sequenced. Choice of control genes and data normalization followed the protocols described in Burton et al. (2008) and Jha et al. (2010). Four control genes were selected: Cyclophilin (AT2G36130), Tubulin alpha-2 chain (TUA2, AT1G50010), Actin 2 (ACT2, AT3G18780) and Glyceraldehyde 3-phosphate dehydrogenase A (GAPDH, AT3G26650). Four independent biological replicates were used. Two rounds of normalization were performed as shown in Burton et al. (2008) and Jha et al. (2010): (1) in respect to the biological replicates in the same treatments (or of the same transgenic line) using control genes, and (2) in respect to different treatments (or transgenic lines) using control genes. Further normalization relative to control group was performed for **Figure 2B**.

### Two electrode voltage clamp of *xenopus laevis* oocytes

To express *NPF2.5* in *X. laevis* oocytes, a pGEMHE-DEST expression vector (Li et al., 2016) was used. cRNA was prepared using a mMESSAGE mMACHINE kit (Ambion, CA) following the manufacturer's manual. Injection of cRNA into oocytes was performed using a protocol described in Munns et al. (2012). Two electrode voltage clamping was performed following the protocols in Roy et al. (2008). Whole oocyte currents were recorded in ND50 solution (50 mM NaCl, 2 mM K-gluconate, 1.8 mM Ca-gluconate, 1 mM Mg-gluconate and 5 mM HEPES, pH at 7.5, osmolarity 240–260 mOsmol/kg). Data was obtained using pClamp 8.2 software (Axon Instruments).

### Microelectrode ion flux estimations (MIFE)

MIFE set-up and electrode-manufacture were following the methods described in Shabala (2006). MIFE measurement of oocytes were made 2 days after injection. Each oocyte was transferred from Ringer's solution to antibiotic free ND96 solution (93.5 mM NaCl, 2 mM KCl, 1.8 mM CaCl_2_, 2 mM MgCl_2_ and 5 mM HEPES, pH at 7.5, osmolarity 240–260 mOsmol/kg) for 5 s and then transferred to ND48 solution (46.7 mM NaCl, 2 mM KCl, 1.8 mM CaCl_2_, 2 mM MgCl_2_ and 5 mM HEPES, pH at 7.5, osmolarity 240–260 mOsmol/kg) before measurements. Flux measurements were made at animal and vegetal poles for 15 min in both *NPF2.5*- and water-injected oocytes for 15 min following a 2.5 min period of electrode alignment and solution exchange. Net fluxes of 4 to 7 individual oocytes were averaged for each treatment.

MIFE measurements of *npf2.5* plants were made using 7-day old seedlings grown on ½ MS media. Plants were transferred to 48 mM NaCl solution for 30 min before a 15-min long measurement. Net fluxes of 6 to 9 individual seedlings were averaged for each genotype.

### Generation of *NPF2.5* amiRNA knockdown lines

Web MicroRNA Designer (http://wmd3.weigelworld.org/cgi-bin/webapp.cgi) (Schwab et al., 2006) was used to design the microRNA constructs to specifically knockdown *NPF2.5* expression. A 21-bp target sequence was identified from the *NPF2.5* sequence. A set of primers (Supplementary Table 1) was used to incorporate the 21-bp amiRNA sequence into the MIR319a vector (Schwab et al., 2006). The amiRNA construct then was cloned into *pCR8* and transferred to *pTOOL2* (Roy et al., 2013) by a LR reaction (Invitrogen) for constitutive over-expression. The construct was transformed into Arabidopsis Col-0 using an *Agrobacterium*-mediated floral drip method (Weigel and Glazebrook, 2002). Transgenic lines were selected by application of 120 mg/L herbicide glufosinate (Bayer Crop Science, Australia).

### Generation of *NPF2.5* over-expression lines

To clone *NPF2.5*, a cDNA library was prepared by performing a reverse transcription (Superscript III, Invitrogen CA) on total RNA extracted from Arabidopsis roots using TRIzol reagent (Invitrogen, CA). The coding sequence of *NPF2.5* was amplified from cDNA with Platinum *Taq* (Invitrogen, CA) using a forward primer (5′ ATGGCTGATTCAAAATCTGGTG 3′) and a reverse primer (5′ AGGAAAACAACATGTTGTGTCC 3′). After Sanger sequencing to confirm the amplified DNA sequence, *NPF2.5* was cloned into the Gateway® enabled pCR8 entry vector (Invitrogen, CA) and transferred from pCR8 to the pTOOL2 destination vector (Qiu et al., 2016) using LR Clonase II (Invitrogen, CA). The pTOOL2 expression vector was transformed into Arabidopsis Col-0 plants using an *Agrobacterium*-mediated floral dip method (Weigel and Glazebrook, 2002).

### GFP (green fluorescent protein) constructs, plants, and visualization

To determine the subcellular localisation of *NPF2.5*, the coding sequence of *NPF2.5* was transferred to the destination vector *pMDC44* (Curtis and Grossniklaus, 2003) using LR Clonase II (Invitrogen, CA). The vector *pMDC44* containing 5′ *GFP6* was used for generating a *GFP::NPF2.5* construct that was transformed into Col-0 Arabidopsis plants using the floral dip method. Two independent transformations were performed to generate two independent lines. T_2_ transgenic plants were geminated on ½ MS media under hygromycin selection (25 μg/ml) and grown for 2 weeks. GFP fluorescence was visualized using an LSM5 PASCAL laser-scanning confocal microscope (Carl Zeiss, Jena, Germany) running PASCAL imaging software (version 3.2 SP2, Carl Zeiss) with an excitation of 488 nm and an emission of 505–530 nm. Plasmolysis was conducted on a slide using 10% (w/v) sucrose solution.

### Arabidopsis mesophyll cell transient transformation and visualization

To determine the subcellular localisation of NPF2.5 in Arabidopsis protoplasts, a fusion construct *YFP*(*Yellow Fluorescent Protein*)*::NPF2.5* was generated using the coding sequence of *NPF2.5* and a modified destination vector *pattR-YFP* (gateway enabled) (Subramanian et al., 2006). Protoplasts were isolated from Arabidopsis mesophyll cells and PEG-mediated transient transformation were conducted using the protocols described in Conn et al. (2013). Transformed protoplasts were examined using a LSM5 PASCAL laser-scanning microscope (Carl Zeiss, Jena, Germany) running PASCAL imaging software (version 3.2 SP2, Carl Zeiss). An excitation of 436 nm and an emission of 470–535 nm were used for cyan fluorescent protein (plasma membrane marker), while an excitation of 514 nm and an emission of 535–610 nm were used for YFP fusion protein.

### Promoter GUS fusion

The complete 1.65 kb intergenic region between *NPF2.5* and *NPF2.4* (the last gene upstream) was amplified from gDNA with Platinum *Taq* (Invitrogen, CA) using a forward primer (5′ GACACTAACGTGTTCTGTCCTCGTTTTC 3′) and a reverse primer (5′ GGTAGAGAACAAGATGAACCAGGAGGGCAA 3′). The PCR fragment was cloned into the Gateway® enabled entry vector *pCR8*. The *proNPF2.5* was transferred into *pMDC162* (Curtis and Grossniklaus, 2003) to drive *uidA* expression using LR Clonase II (Invitrogen, CA). The expression vector was transformed into Arabidopsis Col-0 plants using the floral dip method. Two-week old T_2_ *proNPF2.5:uidA* plants were GUS-stained for 1 h (seedlings) or 2 h (flowers and true leaves) following the protocol described in Weigel and Glazebrook (2002) using 0.5 mg/ml X-gluc (5-bromo-4-chloro-3-indolyl glucuronide). To prepare cross-sections of roots of *proNPF2.5:uidA* plants, stained material was embedded, dehydrated and sectioned following a method described in Li et al. (2016). Images were taken of two independent transformation events using a Leica ASLMD compound microscope equipped with a DFC480 CCD camera (Leica microsystem, Wetzlar, Germany).

### Heterologous expression of *NPF2.5* in yeast

*NPF2.5* was cloned into the yeast expression vector *pYES-DEST52* using LR Clonase II (Invitrogen, CA) and transformed to a *S. cerevisiae* strain InvSc2 (Invitrogen, CA) using a LiAc/SS DNA/PEG method (Gietz and Schiestl, 2007). A growth inhibition assay was performed using solid SD media containing 2% (w/v) D-galactose, 2% (w/v) agar and NaBr/KBr at concentrations as indicated. Yeast cells grew to an OD_600_ value of 3.0 (using a UV-160A spectrophotometer, Shimaduzu, Japan) were pelleted and resuspended in MQ water. Liquid cultures were serially diluted in sterile MQ water by four successive 10-fold dilutions. From each dilution, 10 μl was placed on SD media (no uracil). Results are presented for one of two independent transformation events performed, each with three technical replicates. Both experiments had consistent results. Plates were incubated at 28°C for 2–3 days and growth phenotypes were recorded.

### Elemental analysis of plant materials

Whole shoots were harvested, weighed, freeze-dried and ground into a powder. Approximately 10–20 mg of shoot material was digested in 2 ml of 1% nitric acid at 80°C overnight. A Chloride Analyser (Sherwood Scientific model 926, Cambridge, UK) was used to determine Cl^−^ content in prepared samples following the manufacturer's instructions. The ${\text{NO}}_{3}^{-}$ assay described in Kamphake et al. (1967) and a micro plate-reader (BMG LABTECH, Germany) were used to determine ${\text{NO}}_{3}^{-}$ content in prepared sample. A model 420 Flame Photometer (Sherwood Scientific Ltd, Cambridge, United Kingdom) was used to determine Na^+^ and K^+^ content in the samples.

### Statistical analysis

In all hydroponic experiments, the plants were randomly distributed. Anion accumulation and transcript abundance in hydroponically grown plants were analyzed by one-way ANOVA and Tukey tests (*n* ≥ 4). Two-tailed *t*-tests were used to compare results from the MIFE and TEVC experiments, see figure legends for number of replicates. All data was plotted and analyzed in Graphpad Prism 6.

## Results

### *In silico* analysis of *NPF2.5* (AT3G45710)

**Figure 1:**
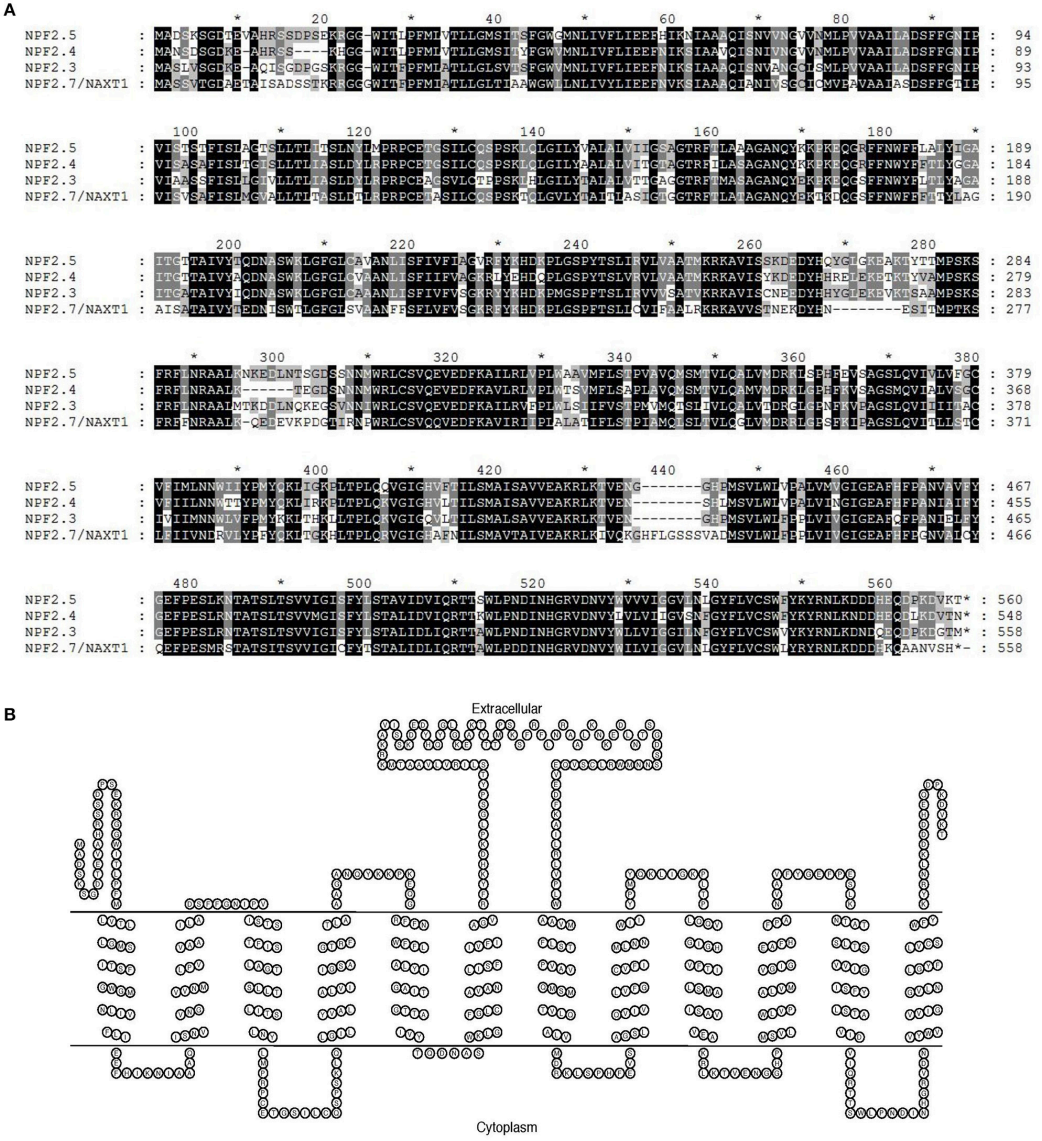
***In silico* analysis of NPF2.5. (A)** Protein sequence alignment of NPF2.5 to NAXT family members with known functions (NPF2.4, NPF2.3, and NPF2.7/NAXT1). Sequence alignment was performed using ClustalW2 with a gap open of 10 and a gap extension of 0.1. Black and shaded regions represent identical residues and conservative substitutions respectively. **(B)** NPF2.5 protein was predicted to have 12 trans-membrane domains and a hydrophobic loop. Trans-membrane domain prediction of NPF2.5 was performed using TMHMM 2.0 with default settings.

The Arabidopsis *NAXT* sub-family members are clustered on chromosome 3 and have high sequence similarity to each other (Segonzac et al., 2007; Tsay et al., 2007). Protein sequence alignment of NPF2.5 to NAXT family members with known functions (NPF2.4, NPF2.3 and NPF2.7/NAXT1) was performed. The NPF2.5 of Col-0 background possesses 83.2% identity (467/561) and 88.6% similarity (497/561) in protein sequence to the Cl^−^ transporter NPF2.4 (Col-0) (Figure 1A). NPF2.5 is predicted to have 560 amino acids, which are predicted to form 12 trans-membrane domains (Figure 1B)-a signature structure of NRT1/PTR proteins (Tsay et al., 2007). A central hydrophilic loop was predicted for NPF2.5, between the trans-membrane domain 6 and 7 (Figure 1B).

### *NPF2.5* is expressed highly in roots and is up-regulated by NaCl treatment

**Figure 2:**
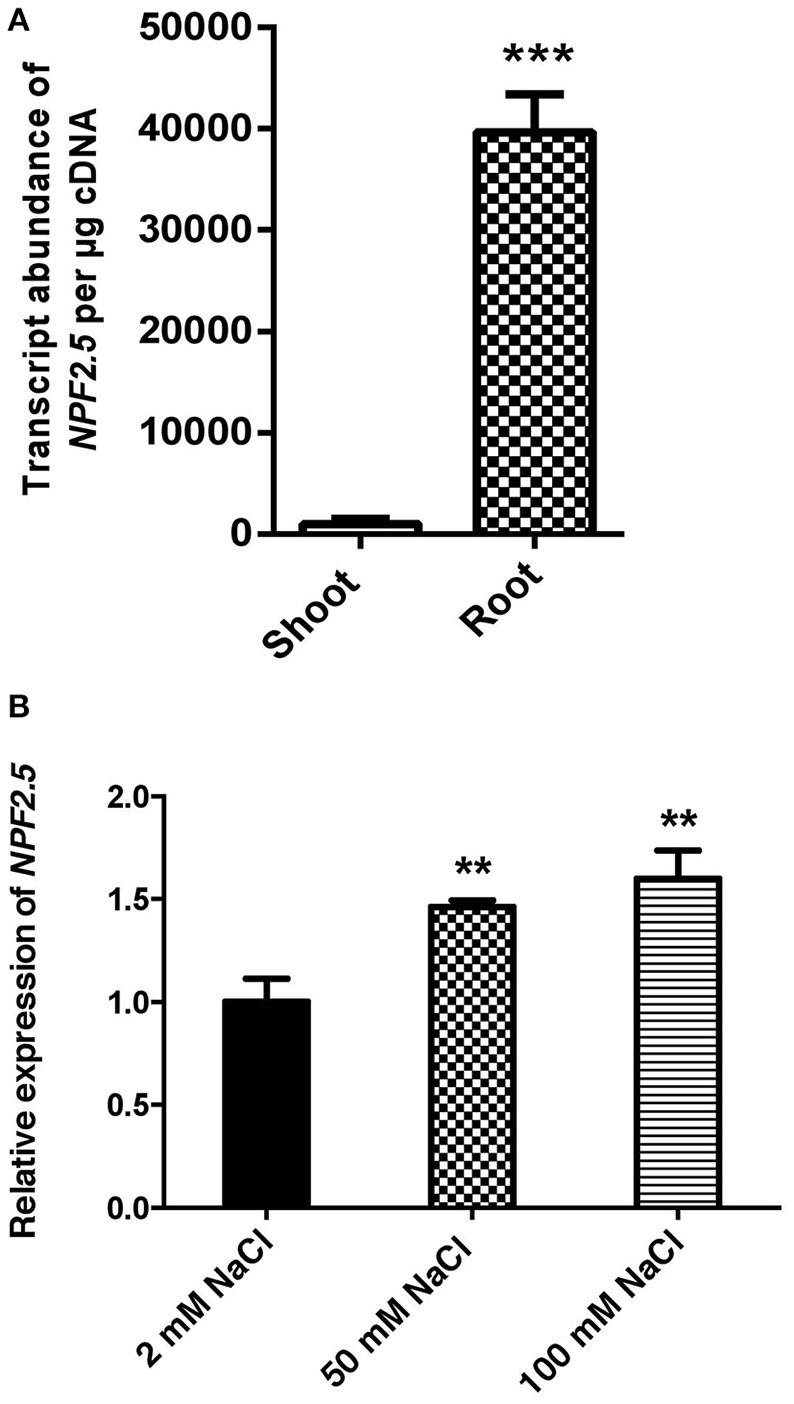
***NPF2.5* is predominantly expressed in the root and is salt-inducible. (A)** *NPF2.5* transcript abundance in the root and the shoot. Col-0 wild type Arabidopsis plants were grown hydroponically for 4 weeks before whole root and shoot were harvested separately for quantitative RT-PCR. **(B)** Transcript abundance of *NPF2.5* was normalized relative to control group (2 mM NaCl) to show expression of *NPF2.5* in response to salt stress. Four-week old Col-0 Arabidopsis plants were grown hydroponically before application of 2 mM, 50 mM or 100 mM NaCl for 72 h. Whole root was harvested for quantitative RT-PCR. Results are presented as mean ± SEM (*n* = 4); significance is indicated by the asterisks (one way ANOVA and Tukey test, ^**^P ≤ 0.01, ^***^P ≤ 0.001).

To determine *NPF2.5* expression in the root and shoot, and its regulation by NaCl salt, quantitative RT-PCR was performed using cDNA prepared from each tissue of 4-week old hydroponically grown Arabidopsis (Col-0) with and without salt treatments. The transcript abundance of *NPF2.5* was 50 times higher in the root than in the shoot when the plants were grown under the low salt condition (2 mM NaCl) (Figure 2A). There was a statistical significant increase in *NPF2.5* transcript abundance in the root when the plants were grown in 50 mM or 100 mM NaCl for 72 h (Figure 2B). At 100 mM NaCl, *NPF2.5* expression increased by 50% compared to that observed in plants grown in 2 mM NaCl (Figure 2B).

### *NPF2.5* is preferentially expressed in root cortical cells

**Figure 3:**
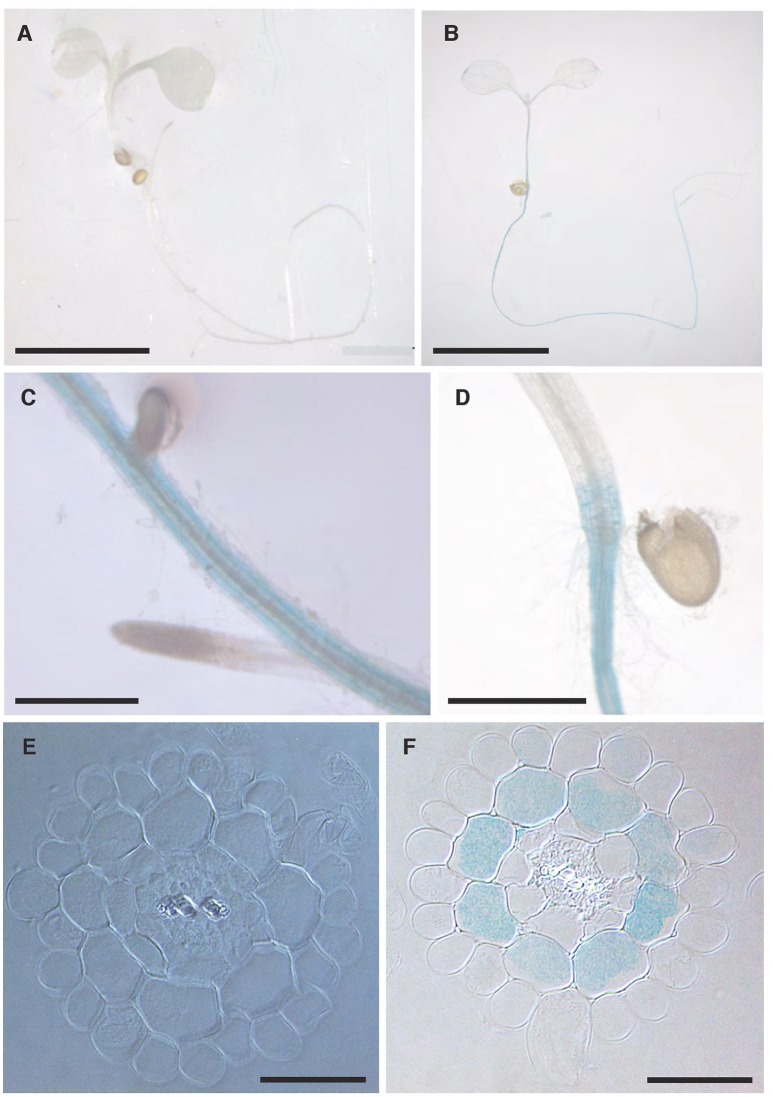
***NPF2*.5 is preferentially expressed in the cortical cells in the root**. T_2_ *proNPF2.5:uidA* plants were grown on ½ MS media for 2 weeks. Plants were harvested and stained for GUS activity for 1 h. **(A)** No GUS activity in a wild type Col-0 plant. **(B)** GUS activity predominantly in the root of *proNPF2.5:uidA* plants. **(C)** GUS activity in the root of *proNPF2.5:uidA* plants. **(D)** GUS activity in the root-to-shoot junction of *proNPF2.5:uidA* plants. **(E)** Root cross-section of non-transformed Col-0 plant. **(F)** Root cross-section of *proNPF2.5:uidA* plant. Scale bars = 4 mm in **(A,B)**, Scale bars = 0.3 mm in **(C,D)**, Scale bars = 0.05 mm in **(E,F)**.

The localisation of *NPF2.5* expression within tissues was determined using the promoter-GUS reporter system. The complete 1.65 kb intergenic region between *NPF2.5* and *NPF2.4* (the last gene upstream) was used to drive the expression of *uidA* in Col-0 Arabidopsis. GUS activity driven by *proNPF2.5* in 2-week old transgenic plants was detected predominantly in the outer cells of the root after 1 h of incubation with X-Gluc (Figures 3B–D). To further determine the specific cell types of the root where *NPF2.5* was expressed, root cross sections of *proNPF2.5:uidA* plants were prepared and revealed that *NPF2.5* was expressed in the root cortex (Figure 3F). To determine if *NPF2.5* was expressed in other tissues at a different developmental stage, flowering *proNPF2.5:uidA* plants (12-week old) were stained for 2 h. GUS activity was detected within stigma, sepals and trichomes of flowering plants (Supplementary Figure 1).

### *NPF2.5* encodes a plasma membrane targeted protein

**Figure 4:**
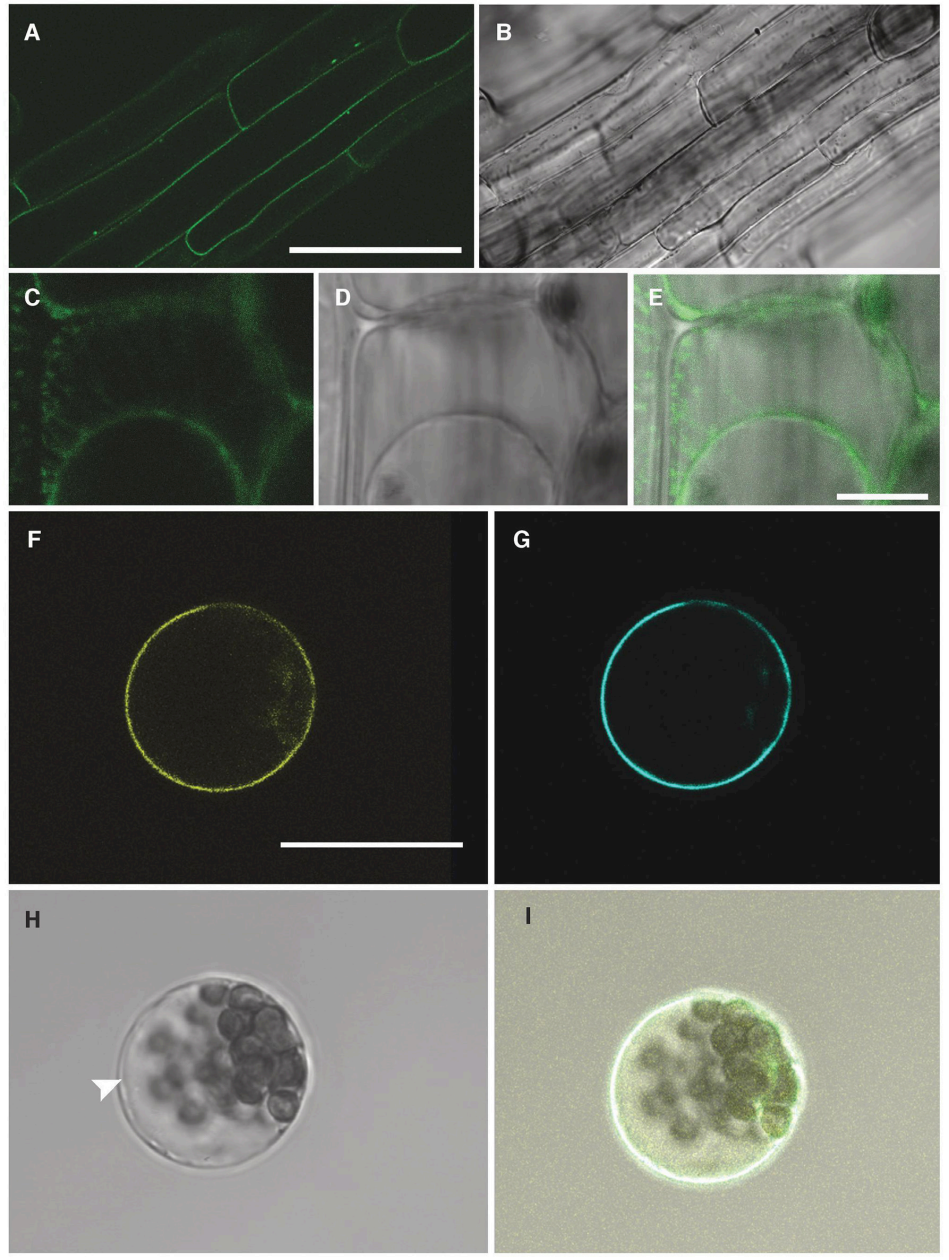
**NPF2.5 is localized to the plasma membrane in Arabidopsis. (A)** Green fluorescence detected in root cells of 2-week old *GFP::NPF2.5* plants. **(B)** Transmitted light image of root cells of 2-week old *GFP::NPF2.5* plants. **(C–E)** Confocal image of plasmolysis performed on root cells of 2-week *GFP::NPF2.5* plants. **(F)** Yellow florescence of YFP::NPF2.5 fusion protein observed in an Arabidopsis mesophyll protoplast. **(G)** Cyan fluorescence of plasma membrane marker eCFP::ROP11 observed in the protoplast. **(H)** Transmitted light image of the protoplast. White arrow points to the separation of the tonoplast from the plasma membrane. **(I)** Merged image showing co-localisation of yellow fluorescence of YFP::NPF2.5 fusion protein and cyan fluorescence of plasma membrane marker. Scale bars = 50 μm in **(A,B)**; Scale bars = 10 μm in **(C–E)**; Scale bars = 20 μm in **(F–I)**.

To determine the sub-cellular localisation of NPF2.5, *GFP* was fused to the 5′ end of the *NPF2.5* coding sequence, and was stably transformed into Col-0 Arabidopsis. *GFP::NPF2.5* plants (T_2_) were grown on ½ Murashige & Skoog (MS) plates for 2 weeks. GFP was detected on the periphery of the root cells of *GFP::NPF2.5* plants (Figures 4A,B). Plasmolysis was used to detach the plasma membrane from the cell wall and revealed GFP fluorescence on the Hechtian strands (Figures 4C–E).

In order to gain further evidence for the membrane localisation of NPF2.5 *in planta, YFP* was fused to the 5′ end of *NPF2.5* coding sequence, and transiently co-transformed with a plasma membrane marker, *eCFP::ROP11* (Molendijk et al., 2008), to isolated Arabidopsis mesophyll protoplasts. Confocal images showed the NPF2.5-associated YFP fluorescence overlapping with the cyan fluorescence of the plasma membrane marker (Figures 4F–I). The position of the tonoplast in the transformed protoplasts is indicated in the bright field image distinct from the plasma membrane (Figure 4H). Therefore, both transformation systems indicated that NPF2.5 resides on the plasma membrane.

### The growth of *NPF2.5* transformed yeast is sensitive to Br^−^, a toxic analog of Cl^−^

**Figure 5:**
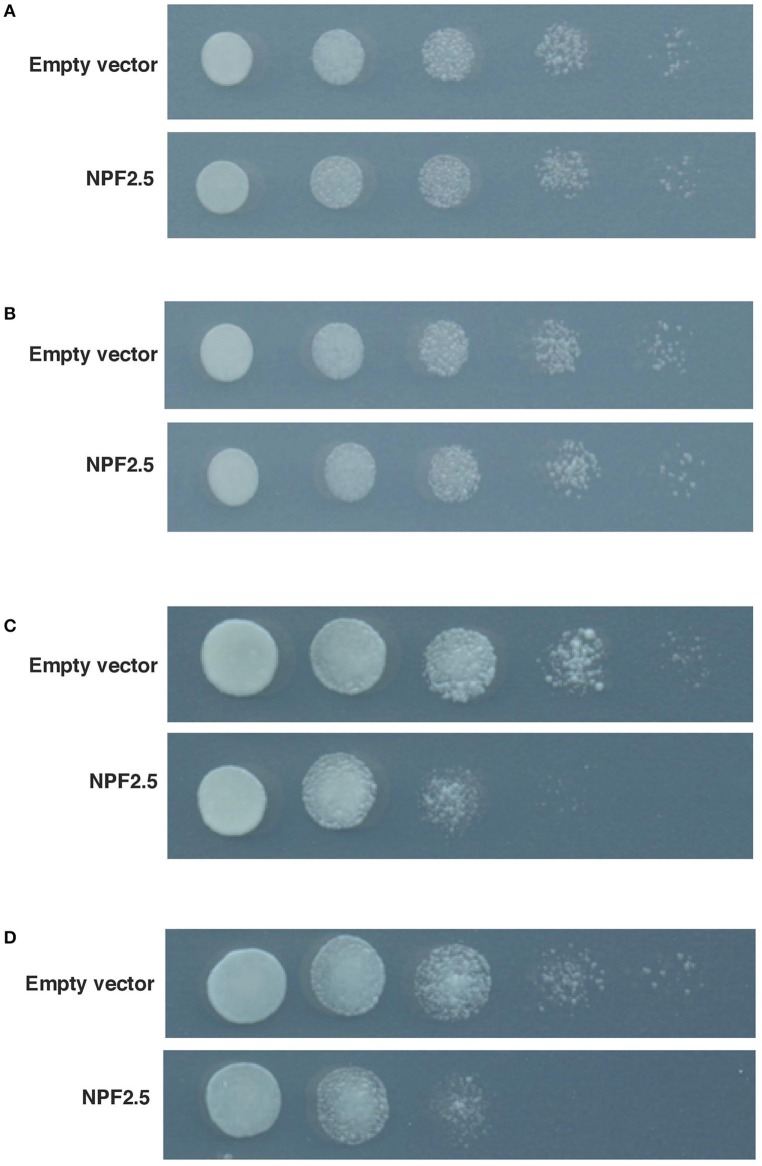
**Growth of *NPF2.5* transformed yeast was inhibited by high levels of external Br^−^**. Yeast was transformed with pYES2-DEST52/*NPF2.5,* or empty vector. Five 10 × serial dilutions of liquid culture were spotted and incubated at 28°C for 2 days on SD medium (-uracil) containing 2% (w/v) galactose, 1.67% (w/v) agar and salts as indicated. **(A)** 500 mM KCl; **(B)** 400 mM NaCl; **(C)** 400 mM KBr; **(D)** 300 mM NaBr.

To test if the NPF2.5 protein was permeable to anions, *NPF2.5* was expressed in yeast (*Saccharomyces cerevisiae*). The transformants were grown on both low and high concentrations of Br^−^, a toxic analog of Cl^−^ (MacRobbie, 1975, 1982). Under low salt conditions (5 mM KBr or NaBr), the growth rate of yeast expressing *NPF2.5* was similar to that of empty vector controls (Figures 5A,B). Under high salt condition (400 mM KBr or 300 mM NaBr), the growth of the *NPF2.5*-transformed yeast was inhibited when compared to the empty vector controls. The inhibition in growth observed was not specific to either K^+^ or Na^+^ (Figures 5C,D). Similar results were obtained across all technical replicates in yeast derived 2 independent transformation events.

### NPF2.5 facilitates Cl^−^ transport in oocytes of *X. laevis*

**Figure 6:**
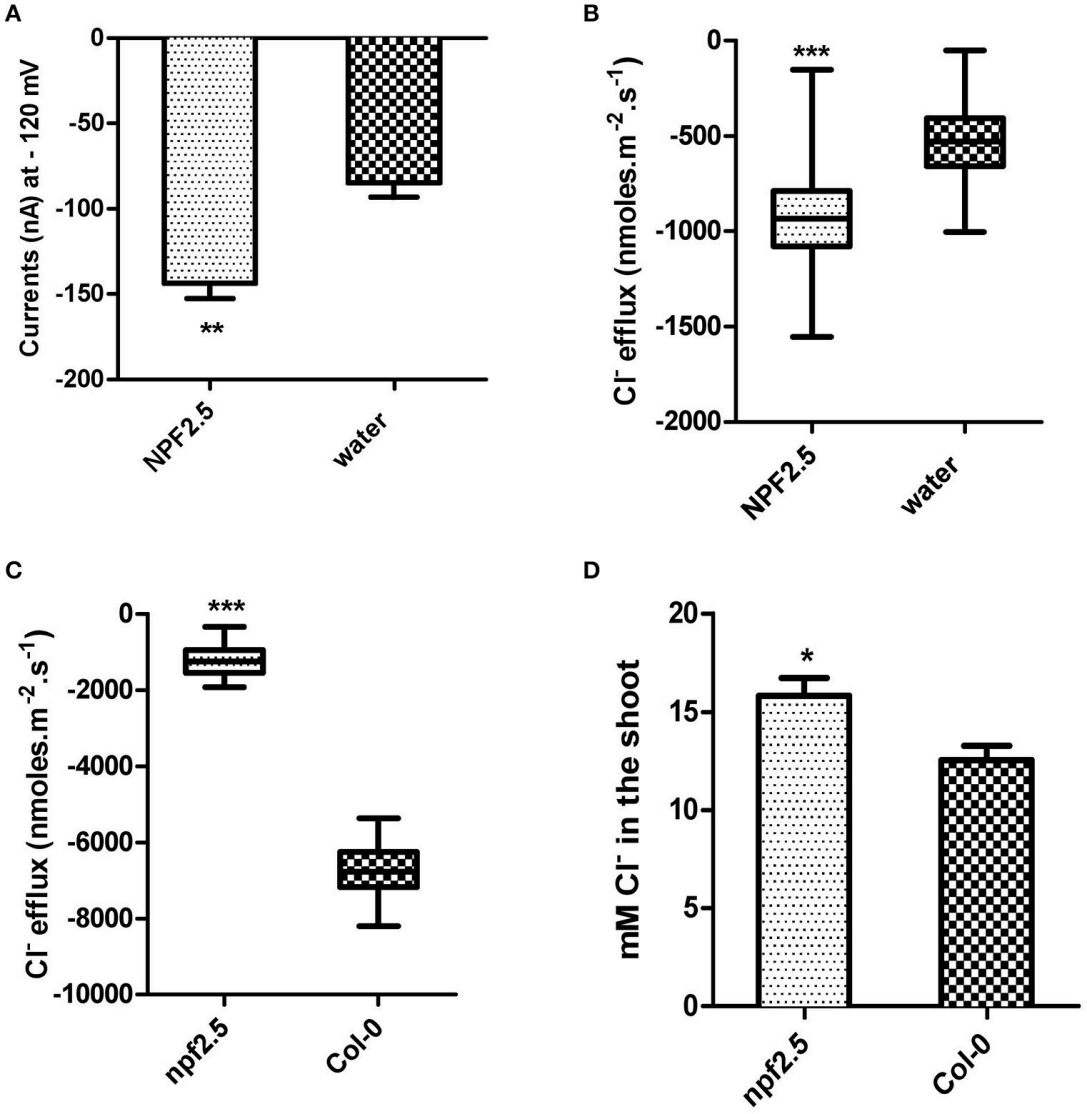
***NPF2.5* encodes a protein that is able to facilitate Cl^−^ efflux**. MIFE and TEVC were performed to study putative Cl^−^ transporter NPF2.5 both *in planta* and in heterologous system. **(A)** Inward currents of oocytes expressing *NPF2.5* when membrane potential was clamped at −120 mV. Oocytes were incubated in a solution of ND50 (50 mM Cl^−^). Results are presented as mean ± SEM (*n* = 7); significance is indicated by asterisks (Two-tailed *t*-test, ^**^P ≤ 0.005) (derived from Supplementary Figure 2A). **(B)** MIFE measurement of *X. laevis* oocytes for Cl^−^ efflux. Oocytes were incubated in ND50 solution. Flux measurements were made for 15 min. Net fluxes of 5 or 7 individual oocytes were averaged for each treatment. Results are presented as mean ± SEM (*n* = 7 for *NPF2.5* injected ones; *n* = 5 for controls; Two-tailed *t*-test, ^***^P ≤ 0.001). **(C)** MIFE measurement of plants for Cl^−^ efflux. 2-week old *npf2.5* and Col-0 plants were transferred to 48 mM NaCl solution for 30 min before a 15-min long measurement. Net fluxes of 6 or 9 individual seedlings were averaged for each genotype. Results are presented as mean ± SEM (*n* = 9 for *npf2.5*; *n* = 6 for Col-0; Two-tailed *t*-test, ^***^P ≤ 0.001). **(D)** *NPF2.5* knockout mutant plants accumulated higher Cl^−^ in the shoot. Four-week old T_4_ *npf2.5* and Col-0 plants were grown hydroponically before being treated with 75 mM NaCl for 5 days. Cl^−^ accumulation in the shoot of *npf2.5* after salt treatment is presented as mean ± SEM (*n* = 9 for *npf2.5*; *n* = 4 for Col-0); significance is indicated by asterisks (Two-tailed *t*-test, ^*^P ≤ 0.05).

To gain further insight into the transport properties of NPF2.5, *NPF2.5* was expressed in *X. laevis* oocytes and two Electrode Voltage Clamp was performed. Significantly higher inward currents were elicited from oocytes pre-injected with *NPF2.5* cRNA when compared with water-injected control oocytes when the membrane potential was clamped at -120 mV (similar to that of plant cells) (Figure 6A); full current-voltage relationships can be found in Supplementary Figure 2. The net Cl^−^ flux across the plasma membrane of *X. laevis* oocytes injected with either *NPF2.5* cRNA or water were compared using non-invasive Microelectrode Ion Flux Estimation (MIFE) (Shabala, 2006). A greater Cl^−^ efflux was observed in *NPF2.5* cRNA injected oocytes than water-injected controls when the [Cl^−^] was lowered from 96 to 48 mM in isosmotic solutions (Figure 6B).

### *NPF2.5* facilitates Cl^−^ efflux from plant roots

To further analyse the putative molecular function of NPF2.5, a *npf2.5* knockout line was obtained. Quantitative RT-PCR was used to confirm the elimination of *NPF2.5* expression in the knockout line (Supplementary Figure 3). MIFE was performed on the *npf2.5* knockout mutant pre-treated with 48 mM NaCl for 5 days. A significantly lower efflux of Cl^−^ from *npf2.5* roots was observed when compared with Col-0 plant roots (Figure 6C).

### Reduced expression of *NPF2.5* led to increased accumulation of Cl^−^ in the arabidopsis shoot under salt stress

The *npf2.5* knockout line was also used to determine whether abolishing *NPF2.5* expression could alter Cl^−^ accumulation in the shoot. When *npf2.5* and Col-0 plants were grown in soil for 4 weeks and treated with either 75 mM or 100 mM NaCl solution for 5 days, shoot Cl^−^ concentration was found to be higher in *npf2.5* plants compared with the Col-0 plants (Figure 6D, Supplementary Figure 4A). There was no difference in accumulation of ${\text{NO}}_{3}^{-}$, K^+^, or Na^+^ in the shoot of *npf2.5* plants when compared to Col-0 plants (Supplementary Figures 4B–D).

**Figure 7:**
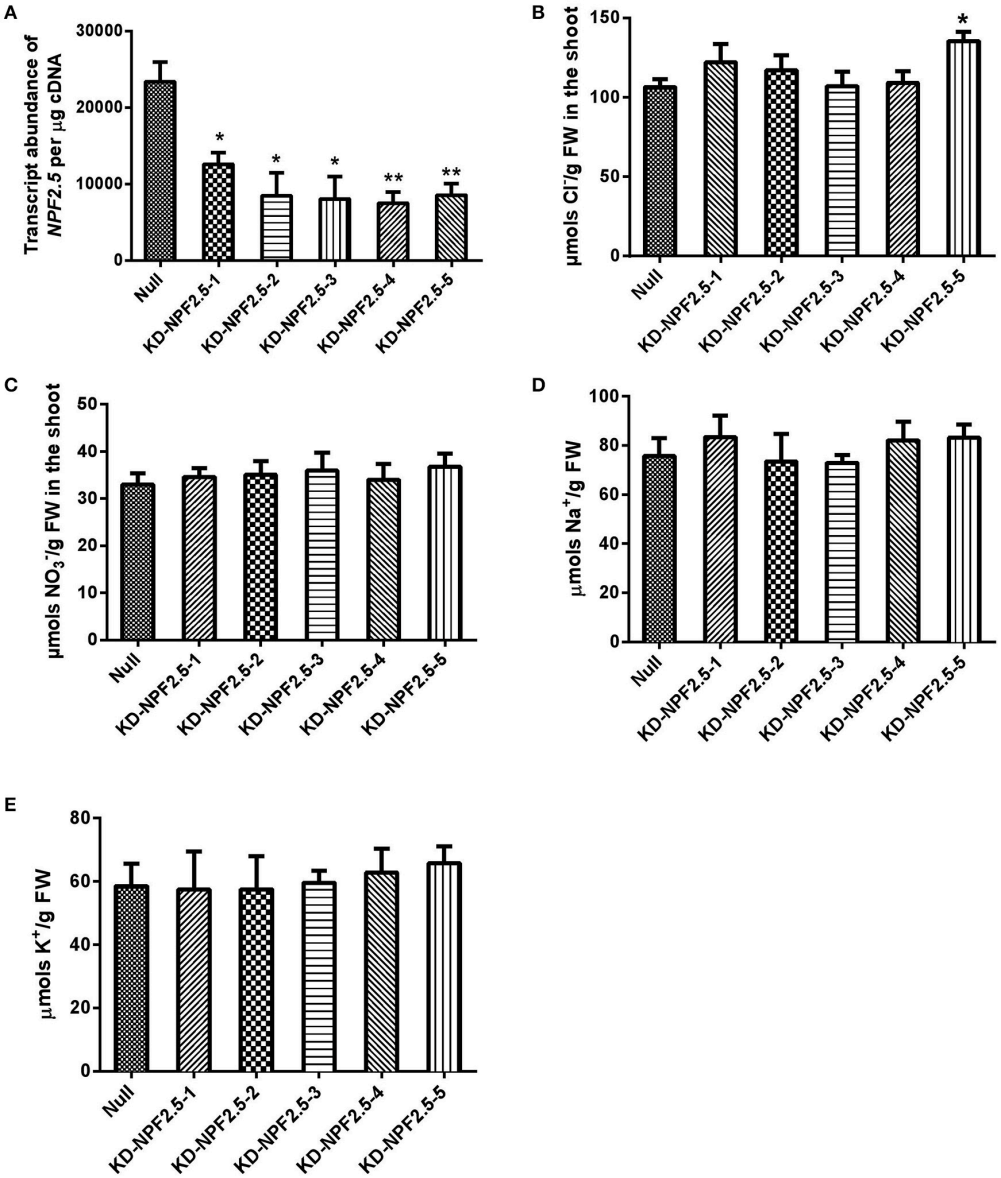
***NPF2.5* amiRNA lines treated with 75 mM NaCl**. Four-week old hydroponically grown T_2_ *NPF2.5* amiRNA lines were treated with 75 mM NaCl for 5 days before harvest. **(A)** *NPF2.5* transcript abundance detected in roots of knockdown lines and null segregate controls. **(B)** Shoot Cl^−^ accumulation in knockdown lines and null segregate controls. **(C)** Shoot ${\text{NO}}_{3}^{-}$ accumulation in knockdown lines and null segregate controls. **(D)** Shoot Na^+^ accumulation in knockdown lines and null segregate controls. **(E)** Shoot K^+^ accumulation in knockdown lines and null segregate controls. Results are presented as mean ± SEM (*n* = 4); significance is indicated by asterisks (one way ANOVA and Tukey test, ^*^P ≤ 0.05; ^**^P ≤ 0.01).

To further test the effect of reduced expression of *NPF2.5* on accumulation of Cl^−^ in the shoot, artificial microRNA (amiRNA) was used to knockdown the expression of *NPF2.5* in the root of Col-0 Arabidopsis. A total of five independent amiRNA-*NPF2.5* knockdown lines were generated (KD-NPF2.5-1, KD-NPF2.5-2, KD-NPF2.5-3, KD-NPF2.5-4, and KD-NPF2.5-5). Transgenic plants were grown hydroponically for 4 weeks and treated with 75 mM NaCl for 5 days. Following salt treatment, the transcriptional levels of *NPF2.5* in the knockdowns were significantly lower than in the null segregants (Figure 7A). Knockdown lines tended to accumulate more Cl^−^ in the shoot compared with the null segregants (*P* = 0.014 for KD-NPF2.5-5) (Figure 7B). Shoot accumulation of ${\text{NO}}_{3}^{-}$, K^+^ and Na^+^ in the knockdown plants was similar to null segregants (Figures 7C–E). To test if the expression of *NPF2.4*, a stelar Cl^−^ transporter with high homology to *NPF2.5*, was affected in the amiRNA *NPF2.5* knockdowns, quantitative RT-PCR was performed on the roots of amiRNA-*NPF2.5* knockdown lines grown in low salt conditions (2 mM NaCl). *NPF2.4* expression of the amiRNA mutants was at the same level as the null segregants (Supplementary Figure 5).

### Constitutive over-expression of *NPF2.5* in arabidopsis affected Cl^−^ accumulation in the shoot under low salt conditions

**Figure 8:**
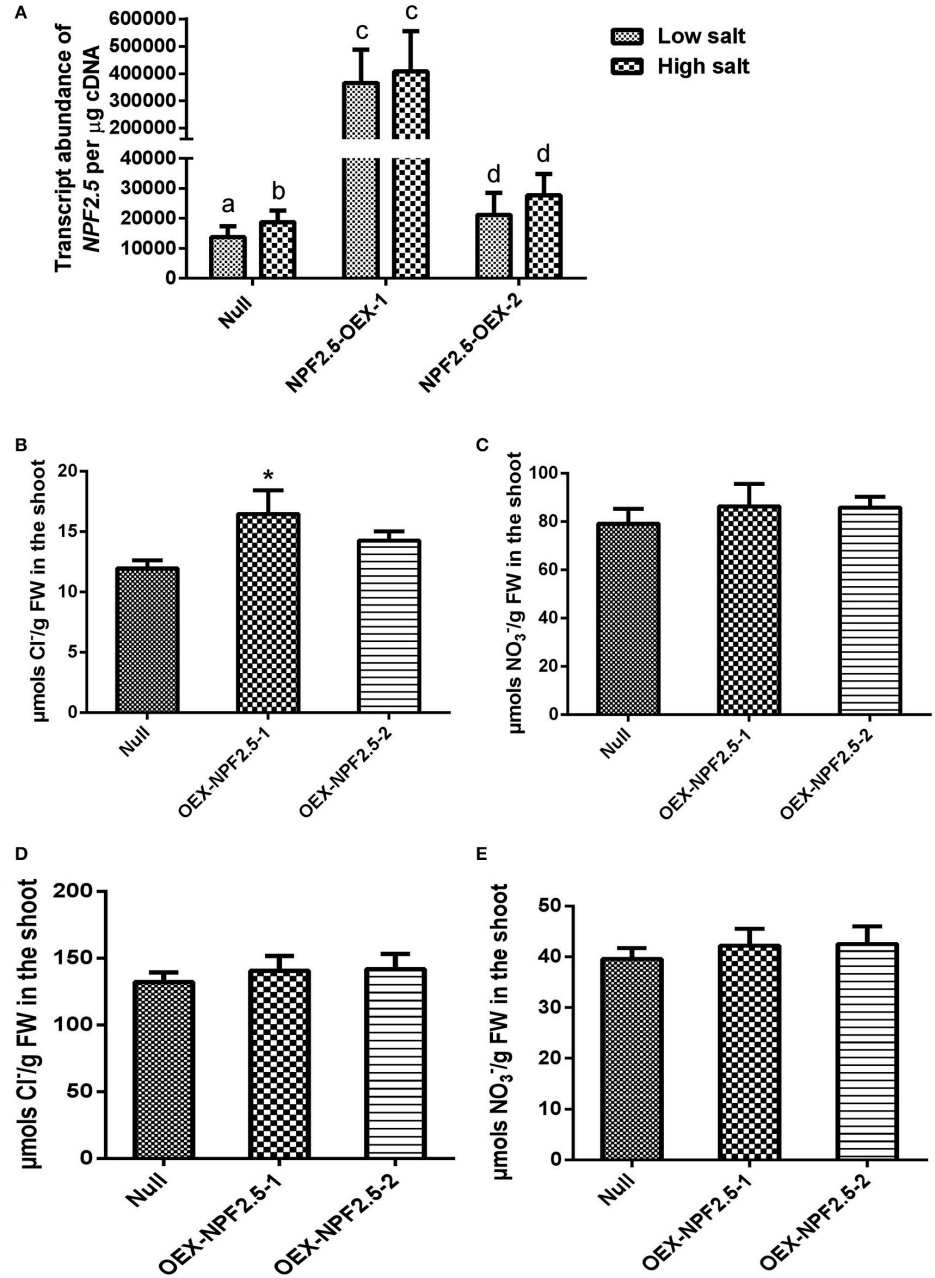
***NPF2.5* over-expression lines treated with low/high salt**. Four-week old hydroponically grown T_3_ *NPF2.5* over-expression lines were treated with 2 mM (low salt) or 75 mM NaCl (high salt) for 5 days before harvest. **(A)** *NPF2.5* transcript abundance detected in roots of mutant lines and null segregant control plants. Columns with different letters indicate statistically significant difference (*P* ≤ 0.05). **(B)** Shoot Cl^−^ accumulation in over-expressing lines and null segregate controls after low salt treatment. **(C)** Shoot ${\text{NO}}_{3}^{-}$ accumulation in over-expressing lines and null segregate controls after low salt treatment. **(D)** Shoot Cl^−^ accumulation in over-expressing lines and null segregate controls after high salt treatment. **(E)** Shoot ${\text{NO}}_{3}^{-}$ accumulation in over-expressing lines and null segregate controls after high salt treatment. Results are presented as mean ± SEM (*n* = 4); significance is indicated by asterisks (one way ANOVA and Tukey test, ^*^P ≤ 0.05).

To determine if constitutive over-expression of *NPF2.5* had an effect on Cl^−^ accumulation, *NPF2.5* was over-expressed in Col-0 Arabidopsis under the control of the *35S* promoter. Two independent T_3_ *NPF2.5* over-expression lines (OEX-NPF2.5-1 and OEX-NPF2.5-2) were grown hydroponically for 4 weeks and treated with 2 mM (low salt) or 75 mM (high salt) NaCl for 5 days. Quantitative RT-PCR revealed that both lines had significantly higher abundance of *NPF2.5* transcript when compared with null segregants, with OEX-NPF2.5-1 plants being approximately 40-fold higher (Figure 8A). Following a low salt treatment, Cl^−^ concentration was higher in the shoot of both over-expression lines when compared with null segregants, with OEX-NPF2.5-1 being statistically significant (Figure 8B). Shoot ${\text{NO}}_{3}^{-}$ concentration in both over-expression lines was shown to be at the similar levels as the null segregants (Figure 8C). After a high salt treatment, shoot accumulation of Cl^−^ and ${\text{NO}}_{3}^{-}$ increased and decreased respectively in all plants tested (Figures 8B–E). However, under these conditions, over-expression lines had similar anion concentrations when compared with null segregants (Figures 8D,E).

## Discussion

NPF2.5 was selected as a putative Cl^−^ transporter due to its sequence similarity to NPF2.4, which had previously been characterized to facilitate loading of Cl^−^ from the root symplast to the xylem (Li et al., 2016). In contrast to *NPF2.4*, expression of *NPF2.5* in the root was found to be specific to the cortex (Figure 3), not the stele, and its transcript abundance increased upon salt treatment (Figure 2), whereas that of *NPF2.4* decreased (Li et al., 2016). Although this does not rule the protein out from a role in anion transport in the roots, these properties are divergent from the predicted criteria for a protein that directly regulates the major loading pathway of Cl^−^ to the root xylem, i.e., it is expressed predominantly in the stele and transcriptionally down-regulated by salt (Li et al., 2016). Regardless, the transport properties of NPF2.5 was examined to explore the putative role of NPF2.5 in the roots, and to examine whether its transcriptional up-regulation by salt has any physiological significance.

Heterologous expression of *NPF2.5* in yeast increased the sensitivity of yeast growth to Br^−^ (Figure 5), this suggests that NPF2.5 has the capacity to transport monovalent anions (MacRobbie, 1975, 1982). Further examination within another heterologous expression system (*X. laevis* oocytes), found that *NPF2.5* cRNA injection induced currents significantly greater in magnitude across the oocyte PM than water-injected oocytes (Figure 6B and Supplementary Figure 2), and that Cl^−^ efflux at resting membrane potentials was also greater from *NPF2.5* injected oocytes (Figure 6A). Both these data are consistent with NPF2.5 being a pathway for Cl^−^ efflux from cells. When net Cl^−^ flux was measured from roots, the magnitude of Cl^−^ efflux from salt treated Col-0 wildtype plants was much greater than that of *npf2.5* knockout mutants (Figure 6C), indicating a role for NPF2.5 in Cl^−^ efflux from Arabidopsis roots. Consistent with this role, the accumulation Cl^−^ in the shoot of *npf2.5* plants was greater than that of Col-0 wildtype plants; furthermore, this occurred without affecting the accumulation of Na^+^, K^+^ or ${\text{NO}}_{3}^{-}$ in the shoot (Figure 6D and Supplementary Figure 4). The observation that Cl^−^ accumulation in the shoot of *npf2.5* grown in 75 mM NaCl was higher than controls by ~10% supports the previous findings that the control of Cl^−^ loading to the shoot is a multicomponent and multigenic trait (Kohler and Raschke, 2000; Gilliham and Tester, 2005; Teakle and Tyerman, 2010; Gong et al., 2011; Li et al., 2016).

Additional independent knockout lines of *NPF2.5* were not available; therefore, amiRNA knockdown plants were generated to further validate the role of NPF2.5 in Cl^−^ transport, and results using these plants were in general consistent with the findings from the knockout line. When the expression of *NPF2.5* in the root was decreased, 3 out of 5 knockdown lines accumulated more Cl^−^ in the shoot (Figure 7B). Notably, KD-NPF2.5-5, one line with reduced expression of *NPF2.5* (by 64%) (Figure 7A), accumulated the most Cl^−^ in the shoot (by 21%) compared to null segregant lines (*P* = 0.014) (Figure 7B). As amiRNA knockdowns did not have complete removal of *NPF2.5* transcripts this may explain why the increases in Cl^−^ concentration in the shoot Cl^−^ of the amiRNA lines were not as great as the *npf2.5* knockout lines over controls.

To further determine whether NPF2.5 was involved in Cl^−^ transport *in planta, NPF2.5* was over-expressed constitutively. Overexpression data from plants should be treated with caution due to common pleiotropic responses-see below, but the fact that both over-expression lines had a Cl^−^ accumulation phenotype in the shoot over the null segregant lines further (Figure 8B) indicates that NPF2.5 is likely to have a role in Cl^−^ transport *in planta*. To understand the physiological role of a particular transporter protein, it is best practice to manipulate its expression specifically in the cells in which it is ordinarily expressed (Møller et al., 2009; Plett et al., 2010; Henderson and Gilliham, 2015). For example, manipulation of the expression of *AtHKT1.1*, a gene encoding a protein that is important for retrieving Na^+^ from the root xylem, results in very different phenotypes depending on the cells in which the expression of the transporter is manipulated (Sunarpi et al., 2005; Møller et al., 2009; Plett et al., 2010). Constitutive over-expression of *AtHKT1.1* resulted in increased Na^+^ accumulation in the shoot, leading to a salt-hypersensitive phenotype (Rus et al., 2004). This is likely to be due to the plants having AtHKT1;1 protein present in outer root cells, a location where the native protein is not ordinarily found, resulting in an increased Na^+^ uptake from the soil. Importantly, cell type specific expression of *AtHKT1;1* in stele, using an enhancer-trap system, improved plant salinity tolerance because of increased removal of Na^+^ from the xylem (Møller et al., 2009). The higher Cl^−^ accumulation found in *NPF2.5* constitutive over-expression lines may result from the presence of NPF2.5 being in every cell type rather than just in the cortex, in particular in the stelar cells where it would not normally be present (Figure 8A). Efflux of Cl^−^ from the stelar symplast into the xylem in the over-expression lines would presumably result in more Cl^−^ in the shoot, not less. Future work should be directed to increase the expression of *NPF2.5* specifically in the root cortical cells, to test whether increased expression of the transporter reduces shoot Cl^−^ and increases salt tolerance.

While Cl^−^ transport was affected in the knockout, knockdown and over-expression lines, ${\text{NO}}_{3}^{-}$ concentration in the shoot of these plants was not affected (Figures 7C, 8C and Supplementary Figure 4B). This suggests that NPF2.5 may be selective to Cl^−^ over ${\text{NO}}_{3}^{-}$, as was proposed for NPF2.4 (Li et al., 2016). Along with its protein location in the outer part of the root and the protein sequence similarity to NPF2.4 (Figure 1A), NPF2.5 is therefore a good candidate for mediating Cl^−^ exclusion from roots and consequently shoots. Seven NAXT members (NPF2.1-NPF2.7) have been suggested to arise from a gene duplication event on chromosome 3 (Segonzac et al., 2007). It is reasonable to hypothesize that the function-uncharacterised NAXT members may also encode anion transporters that are permeable to Cl^−^ or/and ${\text{NO}}_{3}^{-}$. However, the selectivity of these proteins for Cl^−^ and ${\text{NO}}_{3}^{-}$ needs to be determined with care, given that anion selectivity can be changed by a single residue mutation (Wege et al., 2010; Maierhofer et al., 2014), and that NPF2.3 is selective to ${\text{NO}}_{3}^{-}$ (Taochy et al., 2015). A comprehensive elucidation of the physiological roles of all NAXT members would gain interesting insights into functions of the NRT1/PTR family, a family with diverse transport specificities and physiological roles [e.g., transport of Cl^−^, ${\text{NO}}_{3}^{-}$, ABA, and glucosinolates (Tsay et al., 2007; Kanno et al., 2012; Nour-Eldin et al., 2012; Chiba et al., 2015; Li et al., 2016)].

In conclusion, we have shown that *NPF2.5* is expressed in the root cortex and is significantly up-regulated by NaCl, suggesting an involvement of *NPF2.5* in plant salinity tolerance. Given its plasma membrane location and that NPF2.5 facilitates cellular Cl^−^ efflux, NPF2.5 is likely to contribute to root and shoot Cl^−^ exclusion in response to salt stress. However, regulation of Cl^−^ shoot accumulation is seemingly controlled by multiple mechanisms. Therefore, to enhance Cl^−^ exclusion, more players such as NPF2.5, NPF2.4, SLAH1 and CCC should be targeted synergistically, and manipulated in a cell type specific manner.

## Author contributions

BL performed the majority of the experiments and analysis, and drafted the manuscript; JQ conducted TEVC experiments; MJ conducted MIFE experiments; BX performed plasmolysis and associated imaging of the GFP plants; YL assisted with qPCR analysis; GM developed the methods used for cross-sections of Arabidopsis root; SR, MG, and MT conceived and co-supervised the research, co-wrote the manuscript; all authors commented on the manuscript.

## Funding

This work was supported by: the Australian Research Council (ARC) and the Grains Research and Development Corporation (GRDC) to the Australian Centre for Plant Functional Genomics; ARC Centre of Excellence funding [grant number CE140100008] and ARC Future Fellowship [grant number FT130100709] to MG; ARC Discovery grant [grant number DP1095542], GRDC grant [grant number UA00118] and the King Abdullah University of Science and Technology grant to MT; GRDC grant to SR and MG [grant number UA00145]; and China Scholarship Council (CSC) Scholarship [grant number 2008618091] to BL.

### Conflict of interest statement

The authors declare that the research was conducted in the absence of any commercial or financial relationships that could be construed as a potential conflict of interest.

## Supplementary material

Supplementary Figure 1 ***NPF2.5* expression is also present in other tissues. (A)** GUS activity detected in the flower of 12-week old *pNPF2.5:uidA* plants, plant material was GUS stained for 2 h. **(B)** GUS activity detected in trichomes of 12-week old *pNPF2.5:uidA* plants, plant material was GUS-stained for 2 h. Scale bars = 2 mm in **(A)**; Scale bar = 1 mm in **(B)**.

Supplementary Figure 2 **TEVC performed using *X. laevis* oocytes expressing *NPF2.5***. Current-voltage curve of oocytes pre-injected with either *NPF2.5* cRNA or water. Oocytes were incubated in ND50 solution (containing 50 mM NaCl) when the measurements were taken. Results are presented as mean ± SEM (*n* = 5).

Supplementary Figure 3 ***npf2.5* mutant was confirmed to have eliminated expression of *NPF2.5* in the root**. Four-week old T_4_ *npf2.5* and Col-0 plants were grown hydroponically before being treated with 75 mM NaCl for 5 days. *NPF2.5* transcript abundance after salt treatment was detected in the root of both *npf2.5* and Col-0 plants by qRT-PCR. Results are presented as mean ± SEM (*n* = 4); significance is indicated by asterisks (one way ANOVA and Tukey test, ^***^*P* ≤ 0.001).

Supplementary Figure 4 **Accumulation of Cl^−^, ${\text{NO}}_{3}^{-}$, K^+^, and Na^+^ in the shoot of *npf2.5* plants after a 75 mM NaCl treatment**. Four-week old hydroponically grown T_4_ *npf2.5* plants were treated with 75 mM NaCl for 5 days before harvest. **(A)** Shoot Cl^−^ accumulation. **(B)** Shoot ${\text{NO}}_{3}^{-}$ accumulation. **(C)** Shoot K^+^ accumulation. **(D)** Shoot Na^+^ accumulation. Results are presented as mean ± SEM (*n* = 4).

Supplementary Figure 5 **Transcript abundance of *NPF2.4* in the root of *NPF2.5* amiRNA lines**. Four-week old hydroponically grown T_2_ *NPF2.5* amiRNA lines were gown in normal condition (2 mM NaCl). *NPF2.5* transcript abundance in roots of knockdown lines and null segregate controls. Results are presented as mean ± SEM (*n* = 4).

Supplementary Table 1 **Primers used for overlapping PCR to replace miRNAs in MIR319a with amiRNAs that are specific to knockdown NPF2.5 expression in Arabidopsis**. Overlapping regions are in block letters while complementary regions (complementary to the DNA sequences flanking the overlapping region on the plasmid) are in lower case.

## Abbreviations

ABA: Abscisic Acid
amiRNA: artificial micro-RNA
CCC: Cation-Cl^−^ Co-Transporter
CFP: Cyan Fluorescent Protein
GFP: Green Fluorescent Protein
CLC: Chloride Channel
GUS: β-glucuronidase
HKT: High-Affinity Potassium Transporter
MIFE: Microelectrode Ion Flux Estimations
NAXT: Nitrate Excretion Transporter
NPF: Nitrate Transporter 1/Peptide Transport Family
NRT1/PTR: Nitrate Transporter 1/ Peptide Transporter
SLAC1: Slow Anion Channel Associated 1
SLAHs: SLAC1 Homologues
TEVC: Two Electrode Voltage Clamping
uidA: beta-D-glucuronidase
YFP: Yellow Fluorescent Protein.
