# Supplementary figures and images for: AtNPF2.5 Modulates Chloride (Cl−) Efflux from Roots of Arabidopsis thaliana
Source: Front Plant Sci. 2017 Jan 5;7:2013. doi: 10.3389/fpls.2016.02013 (PMC5216686; doi:10.3389/fpls.2016.02013)

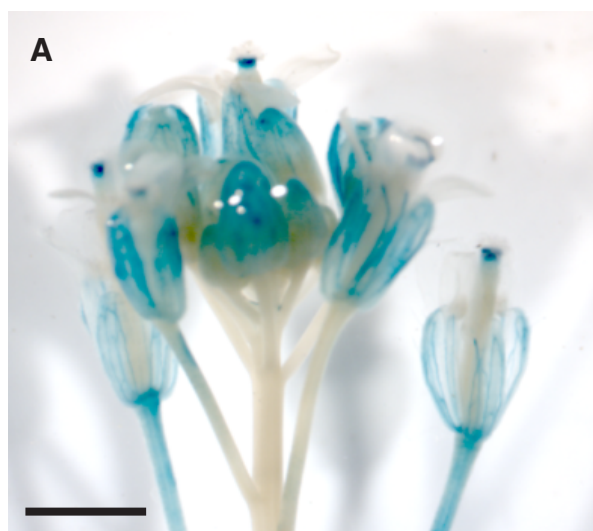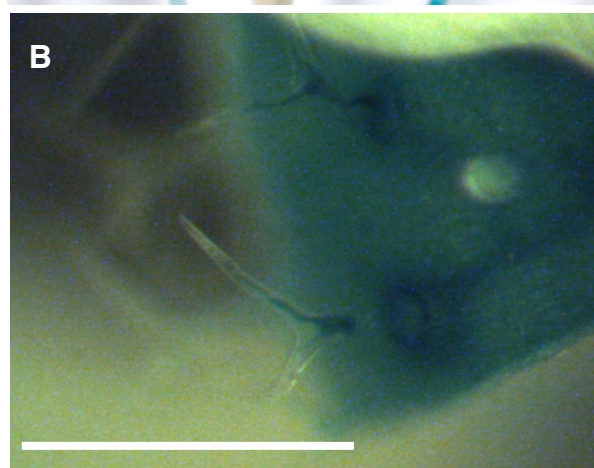

Supplement: Supplementary Figure 1 — NPF2.5 expression is also present in other tissues. (A) GUS activity detected in the flower of 12-week old pNPF2.5:uidA plants, plant material was GUS stained for 2 h. (B) GUS activity detected in trichomes of 12-week old pNPF2.5:uidA plants, plant material was GUS-stained for 2 h. Scale bars = 2 mm in (A); Scale bar = 1 mm in (B). [file Image1.PDF]

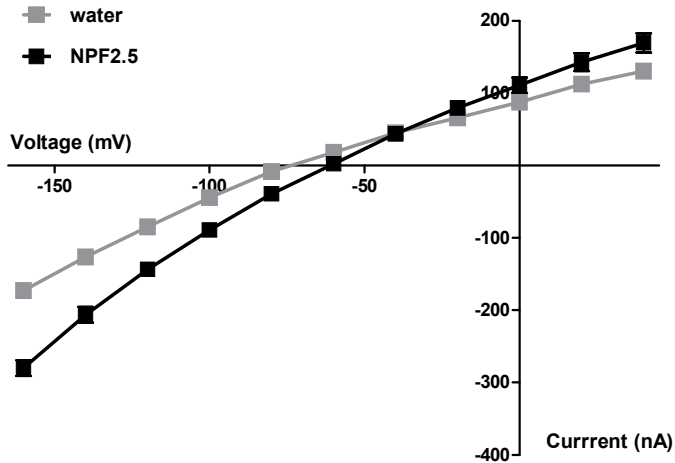

Supplement: Supplementary Figure 2 — TEVC performed using X. laevis oocytes expressing NPF2.5. Current-voltage curve of oocytes pre-injected with either NPF2.5 cRNA or water. Oocytes were incubated in ND50 solution (containing 50 mM NaCl) when the measurements were taken. Results are presented as mean ± SEM (n = 5). [file Image2.PDF]

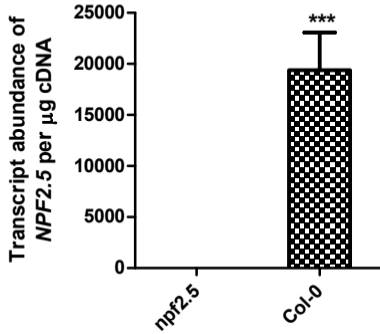

Supplement: Supplementary Figure 3 — npf2.5 mutant was confirmed to have eliminated expression of NPF2.5 in the root. Four-week old T4 npf2.5 and Col-0 plants were grown hydroponically before being treated with 75 mM NaCl for 5 days. NPF2.5 transcript abundance after salt treatment was detected in the root of both npf2.5 and Col-0 plants by qRT-PCR. Results are presented as mean ± SEM (n = 4); significance is indicated by asterisks (one way ANOVA and Tukey test, ***P ≤ 0.001). [file Image3.PDF]

A

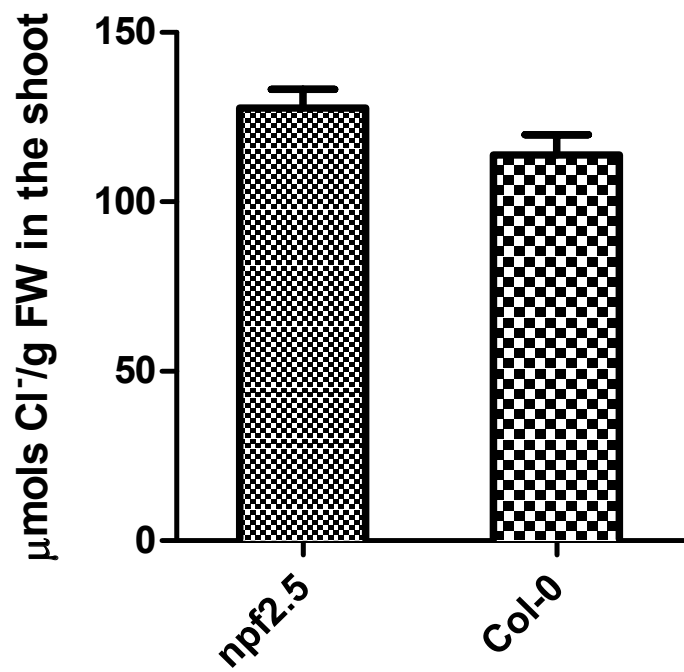

B

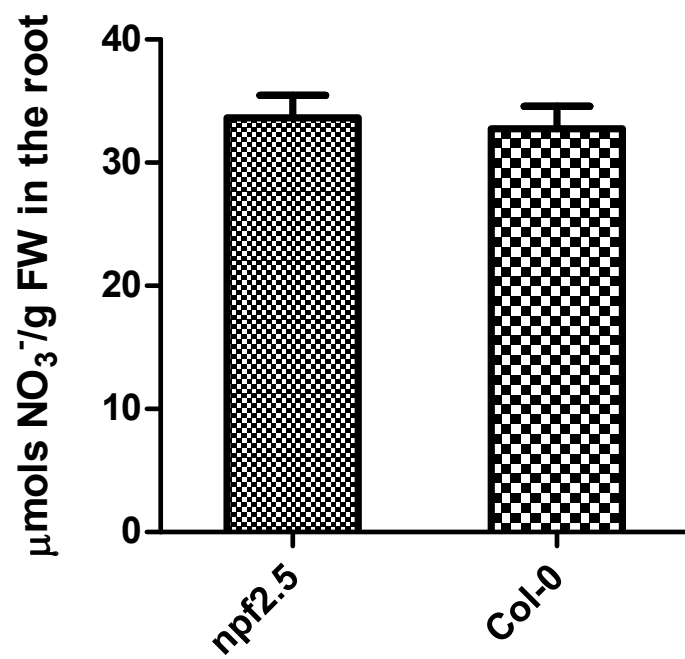

C

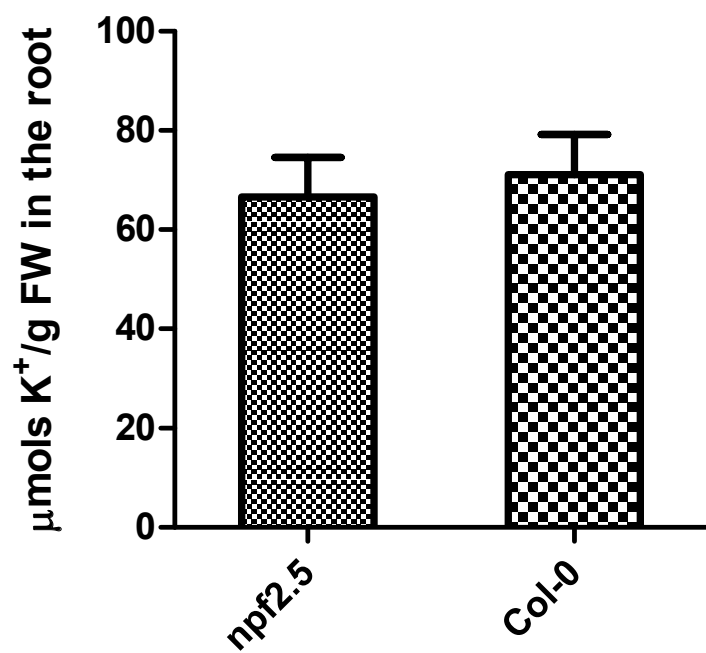

D

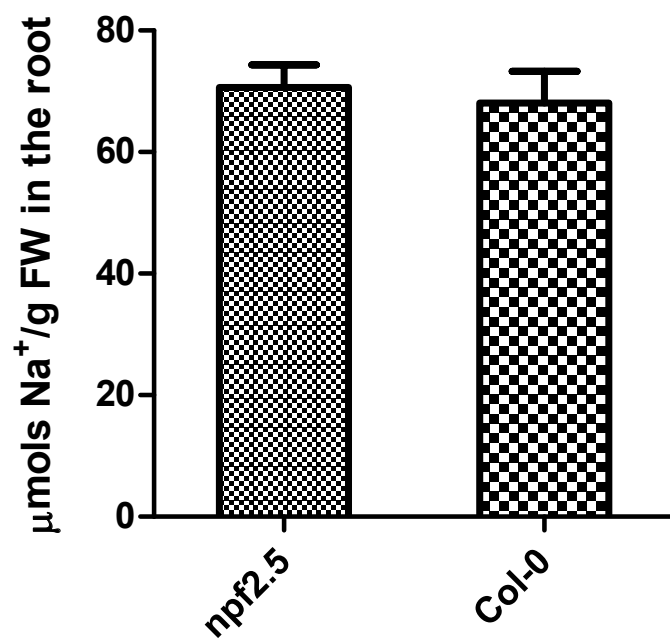

Supplement: Supplementary Figure 4 — Accumulation of Cl−, NO3−, K+, and Na+ in the shoot of npf2.5 plants after a 75 mM NaCl treatment. Four-week old hydroponically grown T4 npf2.5 plants were treated with 75 mM NaCl for 5 days before harvest. (A) Shoot Cl− accumulation. (B) Shoot NO3− accumulation. (C) Shoot K+ accumulation. (D) Shoot Na+ accumulation. Results are presented as mean ± SEM (n = 4). [file Image4.PDF]

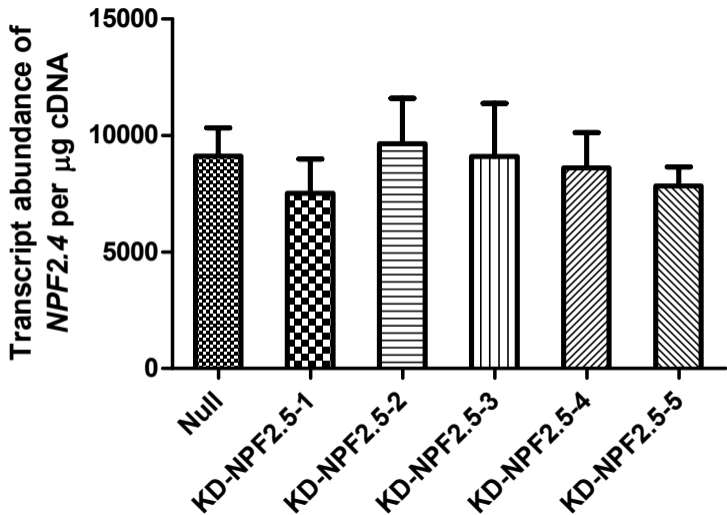

Supplement: Supplementary Figure 5 — Transcript abundance of NPF2.4 in the root of NPF2.5 amiRNA lines. Four-week old hydroponically grown T2 NPF2.5 amiRNA lines were gown in normal condition (2 mM NaCl). NPF2.5 transcript abundance in roots of knockdown lines and null segregate controls. Results are presented as mean ± SEM (n = 4). [file Image5.PDF]
