# Supplementary material for: AtNPF2.5 Modulates Chloride (Cl−) Efflux from Roots of Arabidopsis thaliana
Source: Front Plant Sci. 2017 Jan 5;7:2013. doi: 10.3389/fpls.2016.02013 (PMC5216686; doi:10.3389/fpls.2016.02013)
Supplement: Supplementary Table 1 — Primers used for overlapping PCR to replace miRNAs in MIR319a with amiRNAs that are specific to knockdown NPF2.5 expression in Arabidopsis. Overlapping regions are in block letters while complementary regions (complementary to the DNA sequences flanking the overlapping region on the plasmid) are in lower case. [file Table1.DOCX]

**Supplementary Table 1** Primers used for overlapping PCR to replace miRNAs in MIR319a with amiRNAs that are specific to knockdown *NPF2.5* expression in Arabidopsis.

Overlapping regions are in block letters while complementary regions (complementary to the DNA sequences flanking the overlapping region on the plasmid) are in lower case.

| Target Sequence | Primer name | Primer Sequence |
| --- | --- | --- |
| TAGTATAAGTCTTGGCCCCTT | I miR-s | gaTAGTATAAGTCTTGGATCCCCTTtctctcttttgtattcc |
|  | II miR-a | gaAAGGGGCCAAGACTTATACTAtcaaagagaatcaatga |
|  | III miR-s | gaAAAGGGCCAAGACATATACTTtcacaggtcgtgatatg |
|  | IV miR-a | gaAAGTATATGTCTTGGATCCCTTTtctacatatatattcct |
